# Topology identification and dynamical pattern recognition for Hindmarsh–Rose neuron model via deterministic learning

**DOI:** 10.1007/s11571-022-09812-3

**Published:** 2022-05-13

**Authors:** Danfeng Chen, Junsheng Li, Wei Zeng, Jun He

**Affiliations:** 1 School of Mechatronic Engineering and Automation, Foshan University, Foshan, 528225 People’s Republic of China; 2 School of Physics and Mechanical and Electrical Engineering, Longyan University, Longyan, 364012 People’s Republic of China

**Keywords:** Hindmarsh–Rose neural network, Topology identification, Deterministic learning, Neuronal synchronization, Pattern recognition

## Abstract

Studies have shown that Parkinson’s, epilepsy and other brain deficits are closely related to the ability of neurons to synchronize with their neighbors. Therefore, the neurobiological mechanism and synchronization behavior of neurons has attracted much attention in recent years. In this contribution, it is numerically investigated the complex nonlinear behaviour of the Hindmarsh–Rose neuron system through the time responses, system bifurcation diagram and Lyapunov exponent under different system parameters. The system presents different and complex dynamic behaviors with the variation of parameter. Then, the identification of the nonlinear dynamics and topologies of the Hindmarsh–Rose neural networks under unknown dynamical environment is discussed. By using the deterministic learning algorithm, the unknown dynamics and topologies of the Hindmarsh–Rose system are locally accurately identified. Additionally, the identified system dynamics can be stored and represented in the form of constant neural networks due to the convergence of system parameters. Finally, based on the time-invariant representation of system dynamics, a fast dynamical pattern recognition method via system synchronization is constructed. The achievements of this work will provide more incentives and possibilities for biological experiments and medical treatment as well as other related clinical researches, such as the quantifying and explaining of neurobiological mechanism, early diagnosis, classification and control (treatment) of neurologic diseases, such as Parkinson’s and epilepsy. Simulations are included to verify the effectiveness of the proposed method.

## Introduction

Complex dynamical networks (Chen 2016; Zhang and Wang 2020), whether natural or artificial, are widely exist in almost all scientific and technological fields and play an important role in our lives. Among all kinds of networks, biological neural network is a persistent and hot topic. In the past decade, a lot of studies have been devoted to the synchronization of all kinds of neural networks under different conditions (Li et al. 2003; Cao and Lu 2006; Yu et al. 2009; Ehrich et al. 2013; Fan et al. 2018; Kong and Sun 2021; Wouapi et al. 2020) because of studies have shown that Parkinson’s, epilepsy and other brain deficits are caused by the damage to the ability of neurons to synchronize with their neighbors. As shown in Njitacke et al. (2021), the synchronization of neurons under different structures and parameters were conducted according to the state estimation error system and the control strategy. In Wouapi et al. (2020), the problem of synchronization control of two Hindmarsh–Rose (HR) neuron models was considered, during which, a suitable electronic circuit was designed and applied to the synchronization of two HR systems in the view of Hopf bifurcation control. In the latest study (Zheng et al. 2022), the identification of the unknown topology and parameters of the fractional-order complex dynamical networks was considered based on synchronization and adaptive control, in which, specific controllers and update laws were designed to prompt complete outer-synchronization along with identification.

Besides, topology identification, recognition as well as control of biological neural networks are interesting problems (Yu et al. 2006; Tang et al. 2007; Zhou et al. 2009; Wu et al. 2017; Zhu et al. 2018; Mei et al. 2018; Li et al. 2020; Waarde et al. 2020; Fang et al. 2020; Dong and Zhu 2021), which are significant to some related diseases, such as Parkinson’s and epilepsy. The identification and recognition of neural topology and dynamics are helpful to understand the mechanism of dynamic behavior and signal exchange processes of the neurobiological networks. As presented in Mei et al. (2018), the authors focused on the problem of structure identification for multilayer networks. By using the compressive sensing and regularization, it successfully handled the challenging of structure identification of two layer networks even with noisy observations, which can be further applied to a variety of natural complex systems. Effective control measures of the nervous system help to lay the theoretical foundation for clinical treatment of neurological diseases (Dong and Zhu 2021). Early in 2006, Yu et al. (2006) first considered the problem of topology estimation of networks by using the synchronization method. But the method was valid only under the condition that the local dynamics and coupling functions of each node are almost known precisely (Yu 2010), which is almost impossible for real dynamic systems. In Yang et al. (2020), the authors discussed the energy dependence on discharge models of lzhikevich neuron according to the Hellholes theorem. It was found that the hamiltonian energy of different discharge models is obviously different. However, for precise identification and monitoring of different discharge states, more refined indicators and information are needed. Besides, scholars have done a lot of research on the identification of the dynamical behaviors of neuron models according to the change of discharge behaviors from single-period discharge to double-period discharge, and further to chaotic burst discharge (Wu et al. 2016). Meanwhile, the Lyapunov exponent, bifurcation diagrams, and other indexes also used to analyze the state of neurons. However, for those methods, a great deal of numerical calculations are needed and are only valid if the system parameters are known. Due to the high nonlinearity and complexity of the neural networks, the parameters or the topology of most real-world networks are actually unknown and also difficult to identify exactly (Waarde et al. 2020). In addition, the identification problem becomes more challenging when considering the unknown dynamic environment in which the neural network is located.

In Zhou et al. (2009), authors studied the problem of identification of the topology of a coupled FitzHugh-Nagumo (FHN) neurobiological network via a pinning mechanism. They presented a criterion by using an adaptive feedback controlling method based on Schur complement and Lyapunov stability theory. Similar research was conducted by Zhu et al. (2018), in which, the identification of partial topology of complex dynamical networks was considered, the network synchronization theory and the adaptive feedback controlling method were introduced to reduce the number of neuron nodes. However, for most of the existing studies mentioned above, the linear independent condition which is important for the effectiveness of topology identification has been ignored. Unfortunately, synchronous firing between neurons is inevitable in biological neural networks (Li et al. 2020). In view of this fact, Li et al. (2020) proposed a method that can ensure the linear independence condition and can achieve the identification of the unknown topology of HR Neural Networks.

In addition to the linear independent condition, the persistent excitation (PE) condition (Nar and Sastry 2019) which is significant to the convergence of system parameters and the accurate identification of the unknown system topologies is normally required for system identification. The PE condition can be defined as an intrinsic property of a class of signals. The property is closely related to the exponential stability of a class of linear time-varying systems. For most stability analysis problems of the closed-loop control system, the input signal is always required to be persistently exciting (PE) to ensure that the parameter estimates converge to their true values. Precisely, in the study of synchronization by using the state error system, only the convergence of system state can be ensured. To achieve the more valuable dynamic information, the PE condition is needed. But, the satisfaction of the PE condition is very difficult, especially for application systems.

Over the past few decades, system identification based on neural network (NN) have gained popularity due to its universal approximation ability. The PE property of radial basic function (RBF) networks has been studied over (Gorinevsky 1995; Kurdila et al. 2006; Chen et al. 2014; Nar and Sastry 2019). Furthermore, a deterministic learning (DL) algorithm (Wang et al. 2009) devoted to identification, recognition and control of nonlinear dynamical systems under dynamic environment based on RBF networks was proposed, in which the concept of partial PE condition was defined. According to the DL approach (Wang et al. 2006), it has proved that almost any periodic or periodic-like NN input trajectory, as long as the input trajectory stays within the domain lattice, can lead to desired PE property of a regressor subvector consisting of RBFs whose centers are located in a small neighborhood of the input trajectory. Based on the PE property, locally accurate NN identification of the unknown system topology can be achieved by using localized RBF networks even for a Lyapunov unstable system (Yuan and Wang 2011). The identified system topologies and dynamics were further applied to the dynamical modeling of nonlinear systems (Chen et al. 2016a), the bifurcation prediction of power systems (Chen and Wang 2016b), and the heart valve disorder detection from PCG signals (Zeng et al. 2021), and so on.

Inspired by the discussion given above, this paper mainly focuses on the problem of topology identification and the rapid recognition of the HR neural networks under unknown dynamical environment. The main contents and innovations of this paper lies in that: firstly, the complex nonlinear behavior of the HR neuron system is numerically analyzed through the time responses, system bifurcation diagram and Lyapunov exponent under different system parameters. The results demonstrate that the HR system possesses complex dynamic behaviors with the variation of system parameter, meaning that the HR model satisfies the regression property. This is a new angle of view compared with the traditional numerical dynamic analysis of the HR system. Then, the identification of the nonlinear dynamics and topologies of the HR neural networks under unknown dynamical environment is considered. By using the deterministic learning (DL) algorithm, the unknown dynamics and topologies of the HR system are locally accurately identified and stored as well as represented in a constant neural network form due to the convergence of system parameters. Distinguished from most existing researches, we considered the problem of identification of the HR model in dynamical or non-stationary environments, in which the models are mostly dynamical and deterministic by nature. Additionally, the dynamic information we achieved is the deeper system dynamics behind the state information, which is more valuable to actual systems and are good knowledge reserves for recognition, diagnosis, classification and control of nervous system and neurologic diseases. Finally, a fast dynamical pattern recognition method via system synchronization is constructed according to the time-invariant representation of system dynamics and the similarity definition for two different dynamical patterns based on structural stability. In this section, the time-varying dynamical patterns (training patterns) are effectively represented by the locally accurate NN approximations of the unknown dynamics. Then, the time-varying dynamic patterns are presented in a time-invariant and spatially distributed way, which is essential for the rapid recognition of the HR model. The contribution of this part lies in that the early and rapid recognition of abnormal neural activity plays a key role in the early diagnosis treatment of related diseases. Moreover, the achievements of this work will provide more incentives and possibilities for biological experiments and medical treatment as well as other related clinical researches, such as the quantifying and explaining of biological mechanism, the classification, prediction and control(treatment) of neurologic diseases. Simulations are included to verify the effectiveness of the proposed method.

## Dynamical analysis of the HR model

### The Hindmarsh–Rose model

Early in 1984, Hindmarsh and Rose (1984) proposed a simplified model based on the famous Hodgkin-Huxley (HH) system (Hodgkin and Huxley 1952) to describe the mechanism of neural excitability, i.e., the well-known Hindmarsh–Rose (HR) model. It is a three-dimensional model and can reproduce all kinds of dynamic behaviors under different parameter value, which can accurately describe the voltage and current change on the nerve fiber membrane. A thorough and detailed analysis of the dynamic behavior of the model can help obtain a comprehensive understanding of the characteristics and response of the biological system, which is significant to quantifying and explaining biological mechanism as well as predicting physiological phenomena. The mathematical model of the HR neuron is given by

1 $$\begin{aligned} \begin{aligned} {\dot{x}}&=y-ax^{3}+bx^{2}-z+I,\\ {\dot{y}}&=c-dx^{2}-y,\\ {\dot{z}}&=r(s(x-q)-z). \end{aligned} \end{aligned}$$

where *x* is the membrane potential, *y* is the fast current that associated with the fast current and gating dynamics of $Na^+$ or $K^+$ ions and *z* is the slow current corresponding to the dynamics of calcium ($Ca^{2+}$) channel. The parameter *q* is a real constant standing for the resting potential, *I* is the external stimulus and the other parameters *a*, *b*, *c*, *d*, *r*, *s* are positive real constants.

The nervous system has very strong nonlinear characteristics, in particular, the system dynamics and its topological structures are sensitive to the parameter variation, the external input and the dynamic environment. Under different sets of parameter values, the individual HR model can generate different nonlinear characters including static, tonic spiking, periodic bursting and chaotic behavior and so on.

### Dynamic analysis of the HR model

#### Dynamic behaviors of the HR model under parameter *I*

**Fig. 1:**
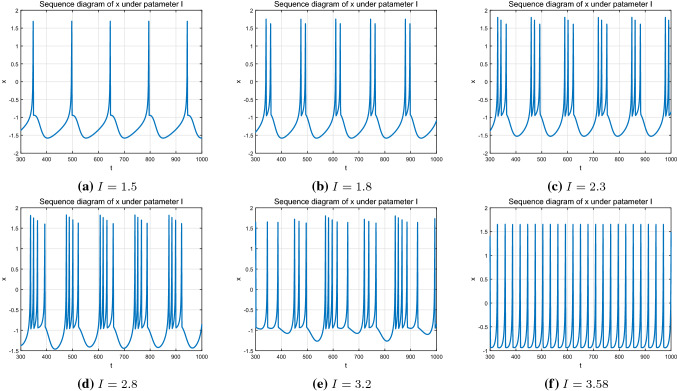
Time response of membrane potential *x* with different parameter *I*

As mentioned above, the HR model given in Eq. (1) is capable of reproducing all the dynamic behaviors exhibited in real biological neuron cells. The external stimulus *I* is taken as the control parameter with the value ranges from 1 to 4, and the other parameters are taken as $a=1.0,b=3.0,c=1.0,d=5.0,r=0.005,s=4.0$ and $q=-1.6$. The initial system state $(x_0,y_0,z_0)$ is set as (0.1, 1.0, 0.2). By varying the parameter *I*, the membrane potential *x* presents different state characteristics, which can be seen from Fig. 1.

Specifically, consider the dynamics of the HR system (1) under variation of the parameter *I*. Set $I=1.5$, the neuron produces regular bursting (single-cycle bursting) as shown in Fig. 1a. When increasing *I* to 1.8, 2.3, 2.8, the system exhibits other kind of regular bursting state, looking as period-doubling, period-3 and period-4 bursting behaviors in turn, which can be seen from Fig. 1b–d, respectively. Further increasing *I* to 3.2, the system becomes chaotic and the topological stability is changed (see Fig. 1e). If increase the value of *I* to 3.58 further, the system returned to a stable single-cycle bursting state as demonstrated in Fig. 1f. Both are single-cycle bursting behaviors, but the period interval shown in Fig. 1f is relatively smaller compared to the period interval shown in Fig. 1a. In addition, the single-cycle trajectory shown in Fig. 1f is relatively regular, which indicates that the corresponding firing process can be relatively stable.

**Fig. 2:**
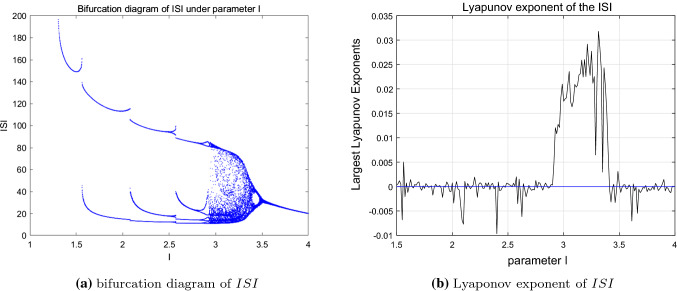
Bifurcation diagram and Lyapunov exponent of *ISI* corresponding to *I*

Besides the time response of the membrane potential *x*, a lot of researches focused on the physiological indicators which are easier to measure and compare them with actual experimental data. The inter-spike interval (*ISI*) is one of the most used physiological indicator. In view of time coding, information is thought to be carried by the *ISI* sequence of neuronal firing. More and more evidences have demonstrated that some neurons encode information through chaotic *ISI* sequence. According to the *ISI* sequence of neurons, the firing patterns of neurons can be divided into periodic and aperiodic discharges [chaotic discharges (Yang and Hu 2002)]. Then, the bifurcation diagram of the (*ISI*) corresponding to each external excitation *I* ranging from 1 to 4 is considered and demonstrated in Fig. 2a. Furthermore, the Lyapunov exponent is another important reference indicator for judging the characteristics of the system dynamics and system stability. The corresponding Lyapunov exponent of the *ISI* sequence under parameter *I* is considered and the diagram is given in Fig. 2b.

The bifurcation and the Lyapunov exponent diagram shown in Fig. 2 indicate that the dynamic characteristics of the HR system shown by the inter-spike interval sequence is consistent with that of the system state discussed in Fig. 1. Starting from $I=1.0$, the HR model experiences period-1,2,3,4 bursting state in turn. During this process, the *ISI* sequence presents a period-adding bifurcation phenomenon. Further increasing the parameter *I* to 3.2, it becomes unstable and at this time, the corresponding *ISI* sequence enters chaotic state. When *I* researches around 3.5, the system state as well as the *ISI* sequence returns to a stable single-period state. According to the Figs. 1 and 2, we can conclude that, the system dynamics and topology of the HR model become more and more complex with the increase of parameter *I* at first, and when it reaches to a critical point, the system reverts back to a simple state. During this process, the topological structure of the system changes from stable to unstable and then to stable. That is, the varying of parameter *I* changes the topological structure of the neuron system, and the system stability is affected accordingly when the structure changes to a certain extent.

#### Dynamic behaviors of the HR model under parameter *r*

**Fig. 3:**
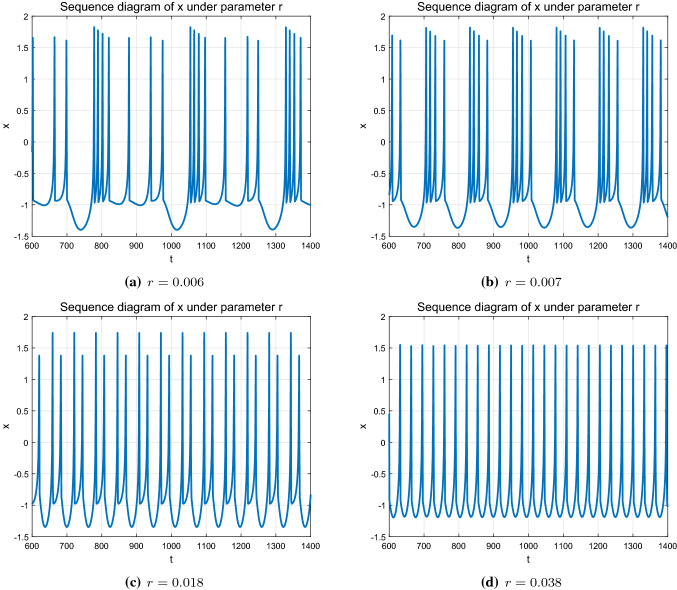
Time response of membrane potential *x* with different parameter *r*

**Fig. 4:**
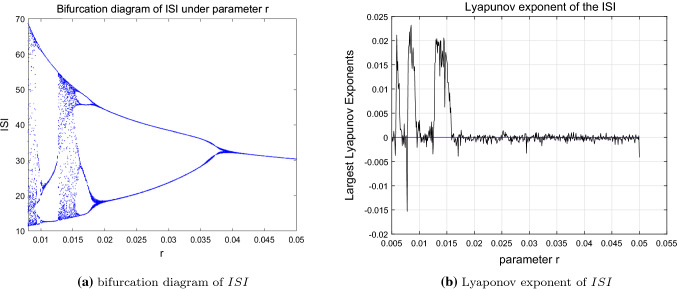
Bifurcation diagram and Lyapunov exponent of *ISI* corresponding to *r*

Besides the external excitation *I*, the parameter *r* given in Eq. (1) is another important parameter, which is related to the calcium $(Ca^{2+})$ concentration and is significant to many neurological disorders (Wu et al. 2017). Different value of *r* can induce different firing patterns of the HR model. In this part, the parameter *r* is taken as the control parameter. All the other parameters are kept as the same as mentioned in “Section Dynamic analysis of the HR model” while parameter *I* is fixed to 3. With the variation of *r* from 0.0 to 0.05, different dynamic behaviors of the HR neural system are presented. The initial state is given as $(x_0,y_0,z_0)=(0.1, 1.0, 0.2)$. As shown in Fig. 3, the time response of membrane potential *x* of the HR model emerges different dynamic behaviors under different *r*. Correspondingly, the *ISI* bifurcation diagram and the Lyapunov exponent with parameter *r* are demonstrated in Fig. 4.

According to the numerical simulation demonstrated in Figs. 3 and 4, the system expresses abundant dynamic behaviors under parameter *r*. Precisely, the state is unstable and chaotic at the beginning ($r=0.006$) and it gradually appears periodic bursting characteristics (period-4 state when $r=0.007$ and periodic-2 state when $r=0.018$) with the increase of the *r*, and it finally reaches to period-1 state when $r \in [0.038,0.05]$. In addition, the system topology and stability also change accordingly from unstable chaotic to regular stable periodic state, which can be seen from the bifurcation and Lyapunov exponent diagrams of the *ISI* under parameter *r* (shown in Figs. 3 and 4).

## Identification of the HR model via deterministic learning theory

The above numerical results are obtained under the condition of given system parameters. However, for most actual nonlinear dynamic systems, especially for those complex systems located in dynamic environment, the parameters are almost unknown. Thus, topology identification under dynamic environment is one of the most important question to be solved, which will obtain a comprehensive understanding of the dynamic characteristics of the HR model. To achieve convergence of system parameters and the accurate identification of system dynamics, the persistent excitation (PE) condition is normally required. However, it is very difficult to characterize or verify the PE condition for nonlinear systems, especially for complex nonlinear systems.

The recently proposed deterministic learning (DL) theory (Wang et al. 2009) present a framework of learning from uncertain dynamic environment. It mainly studies the dynamic process of knowledge acquisition, knowledge expression and knowledge reutilization under dynamic environment. The DL algorithm has proved that almost any periodic or periodic-like (recurrent) NN input can lead to the satisfaction of partial PE condition along the system trajectory under the localized radial basic function (RBF) networks. Based on the above analysis, the DL algorithm is introduced in this part for the identification of the unknown system topology of the HR model.

Consider the HR model given in Eq. (1) as a general nonlinear system given in the following form,

2 $$\begin{aligned} {\dot{x}}=f(x;\mu ),x(t_{0})=x_{0}, \end{aligned}$$

where $x=[x_1,x_2,x_3 ]^T \in R^{3}$ is the state vector of the system. $x_1$ represents the membrane potential (known as *x* as given in the HR system (1)), $x_2$ denotes the fast current of potassium ($K^{+}$) channel (known as *y* of the HR model) and $x_3$ is the slow current of calcium ($Ca^{2+}$) channel (known as *z* of the HR model). $\mu$ is the unknown control parameter that different parameter value in general can produce different dynamical behaviors. $f\;=\;[f_{1}(x;\mu ),f_{2}(x;\mu ),f_{3}(x;\mu )]^T\in R^{3}$ is a smooth but unknown nonlinear function vector.

According to the HR system shown in Eq. (1), it is seen that the HR system is unknown nonlinear but smooth with unknown system parameter and the state *x*, *y*, *z* are uniformly bounded. That is, the state vector *x* given in Eq. (2) satisfies the condition that $x(t)\in \varOmega \subset R^{n}, \forall t\ge t_{0}$, where $\varOmega$ is a compact set. Moreover, the system trajectory starting from point $x(t_{0})=x_{\xi 0}$ denoted as $\varphi _{\xi }(x_{0})$ is in either a periodic or periodic-like (recurrent) motion (Shilnikov et al. 2001).

### Remark 1

A recurrent trajectory can be described as: for given $\xi >0$, there exists constant time $T(\xi )>0$ such that the trajectory returns to the $\xi$-neighborhood of any point to the trajectory within a time *t* ($t\le T(\xi )$). The most striking feature of a recurrent trajectory is that, compared with periodic trajectories, who returns to the original trajectory within a fixed time, the return time for a recurrent trajectory is not fixed but is finite. Specifically, no matter what the initial condition is, given $\xi$, the whole trajectory lies in the $\xi$-neighborhood of the segment of the trajectory corresponding to a bounded time interval $T(\xi )$. This instruction reviews that the recurrent motion contains a wealth of dynamic behaviors including periodic, almost-periodic, quasi-periodic, and even chaotic trajectories. These dynamic behaviors have different structural stability (Shilnikov et al. 2001). It can be seen from “Section Dynamical analysis of the HR model” that the HR model is capable of reproducing all kinds of dynamic behaviors under different parameters including single-cycle busting model, period-doubling model, chaotic model and so on. That’s why we say that the system trajectory starting from point $x(t_{0})=x_{\xi 0}$ denoted as $\varphi _{\xi }(x_{0})$ of the HR model is in either a periodic or periodic-like (recurrent) motion.

For identifying the unknown system dynamics $f(x;\mu )$ of the HR system, the following dynamical radial basic function (RBF) network is considered,

3 $$\begin{aligned} \dot{\hat{x}}=-{\bar{A}}(\hat{x}-x)+\hat{W}^{T}S(x), \end{aligned}$$

where $\hat{x}=[\hat{x}_{1},\hat{x}_{2},\hat{x}_{3}]^T$ is the state vector of the dynamical RBF network model, *x* is the state of system (2). ${\bar{A}}=diag\{{\bar{a}}_{1},{\bar{a}}_{2},{\bar{a}}_{3}\}$ is a diagonal matrix with ${\bar{a}}_{i}$ being a positive design constant. The RBF networks $\hat{W}^TS(x)=[\hat{W}_{1}^TS_{1}(x),\hat{W}_{2}^TS_{2}(x),\hat{W}_{3}^TS_{3}(x)]^T$ are applied for the approximation of the unknown system dynamics $f(x;\mu )=[f_{1}(x;\mu ),f_{2}(x;\mu ),f_{3}(x;\mu )]^T$ given in Eq. (2) within the compact set $\varOmega$. Moreover, for each subsystem, there is $\dot{\hat{x}}_i=-{\bar{a}}_{i}(\hat{x}_{i}-x_{i})+\hat{W}_{i}^TS_{i}(x),i=1,2,3$.

By considering the system given in Eqs.(2) and (3), the derivative of the estimation error of system state ${\tilde{x}}_i=\hat{x}_i-x_i$ can be achieved, that is,

4 $$\begin{aligned} \dot{\tilde{{x}}}_i=-{\bar{a}}_{i}{\tilde{x}}_{i}+\hat{W}_{i}^{T}S_{i}(x)-f_{i}(x;\mu ) =-{\bar{a}}_{i}{\tilde{x}}_{i}+{\tilde{W}}_{i}^{T}S_{i}(x)-\epsilon _i, \end{aligned}$$

where ${\tilde{W}}_{i}=\hat{W}_{i}-W^{*}_{i}$ and $\hat{W}_i$ is the estimate of the ideal weight $W^{*}_{i}$ while $\epsilon _{i}=f_{i}(x;\mu )-W_{i}^{*T}S_i(x)$ is the ideal approximation error. Furthermore, the NN weight estimates $\hat{W}_i$ of the RBF networks are updated by the following learning law:

5 $$\begin{aligned} \dot{\hat{W}}_{i}=\dot{{\tilde{W}}}_{i}=-\varGamma _{i}S_{i}(x)\tilde{{x}}_{i}-\sigma _{i}\varGamma _{i}\hat{W}_{i}, \end{aligned}$$

where $\varGamma _{i}=\varGamma _{i}^T>0$, and $\sigma _{i}>0$ is a small value that can be designed accordingly. $\sigma _{i}\varGamma _{i}\hat{W}_{i}$ is considered as the $\sigma -$ modification part and used to keep the bound of ${\tilde{W}}_{i}$ and $\hat{W}_{i}$.

By considering the PE condition, it requires that the system state *x*(*t*) which is also the input of the RBF network, can visit every center of the whole RBF network “persistently”, which is generally impossible in practice. Thus, the localization property (Wang et al. 2009) of the RBF network is introduced. Precisely, the neurons are divided into two parts according to the distance between the distribution of neurons and the system trajectory $\varphi _{\xi }(x_{0})$, which are denoted as $(\cdot )_{\xi i}$ and $(\cdot )_{{\bar{\xi }} i}$ standing for the regions that close to and away from the trajectory $\varphi _{\xi }(x_{0})$, respectively. Then, Eq. (4) can be expressed in the following form:

6 $$\begin{aligned} \begin{aligned} \dot{\tilde{{x}}}_i&=\ -{\bar{a}}_{i}{\tilde{x}}_{i}+\hat{W}_{\xi i}^{T}S_{\xi i}(x)+\hat{W}_{{\bar{\xi }} i}^{T}S_{{\bar{\xi }} i}(x)-f_{i}(x;\mu )\\&=\ -{\bar{a}}_{i}{\tilde{x}}_{i}+{\tilde{W}}_{\xi i}^{T}S_{\xi i}(x)-\epsilon _{\xi i},\\ \end{aligned} \end{aligned}$$

in which $S_{\xi i}(x)$ is a subvector of $S_{i}(x)$ that corresponding to the neurons distributed close to system trajectory, while ${\tilde{W}}_{\xi i}$ is the corresponding weight subvector. $\epsilon _{\xi i}=\epsilon _{i}+\hat{W}_{{\bar{\xi }} i}^{T}S_{{\bar{\xi }} i}(x)=0(\epsilon _{i})$ is the local approximation error along the trajectory $\varphi _{\xi }(x_{0})$. Since $\hat{W}_{{\bar{\xi }} i}^{T}S_{{\bar{\xi }} i}(x)$ represents the system dynamics identified by neurons located far away from the system trajectory, the corresponding value can be very small. So, $0(\epsilon _{i})$ is considered as a small neighborhood of zero that do close to the global approximation error $\epsilon _{i}$ as given in Eq. (4).

According to the Eqs. (5) and (6), the adaptive system is described as follows:

7 $$\begin{aligned} \left[ \begin{array}{c} \dot{{\tilde{x}}}_{i} \\ \dot{{\tilde{W}}}_{\xi i} \\ \end{array} \right] =\left[ \begin{array}{cc} -{\bar{a}}_{i} &{} S_{\xi i}(x)^{T} \\ -\varGamma _{\xi i}S_{\xi i}(x) &{} 0 \\ \end{array} \right] \left[ \begin{array}{c} {\tilde{x}}_{i} \\ {\tilde{W}}_{\xi i} \\ \end{array} \right] +\left[ \begin{array}{c} -\epsilon _{\xi i} \\ -\sigma _{i}\varGamma _{\xi i}\hat{W}_{\xi i}\\ \end{array} \right] . \end{aligned}$$

Based on the localization property of RBF networks, almost any periodic or recurrent trajectory $\varphi _{\xi }(x_{0})$ starting from the initial point $x_{0}=x(t_{0})\in \varOmega$ under initial weight $\hat{W}_{i}(0)=0$ can ensure the PE condition for those neurons located close to the system trajectory, which is called partial PE condition (Wang et al. 2009). Furthermore, the following conclusions are achieved: 1) the weights of the neurons that located in a local region of the system trajectory will converge to a small neighborhood of their ideal values due to the satisfaction of the partial PE condition, while those that far away from the trajectory will not be activated and the weigh will keep close to zero; 2) the unknown system dynamics can be locally and accurately approximated by using the localized RBF networks along the system trajectory $\varphi _{\xi }(x_{0})$; 3) the learned system dynamics can be stored and represented by a constant vector of neural weights ${\bar{W}}$ due to the convergence of the NN weights. That is,

8 $$\begin{aligned} \begin{aligned} f_{i}(x;\mu )&=\ W^{*T}_{\xi i}S_{\xi i}(x)+\epsilon _{\xi i}=\hat{W}^{T}_{\xi i}S_{\xi i}(x)-{\tilde{W}}^{T}_{\xi i}S_{\xi i}(x)+\epsilon _{\xi i}\\&= \hat{W}^{T}_{\xi i}S_{\xi i}(x)+\epsilon _{\xi i_{1}},\\&= {\bar{W}}^{T}_{\xi i}S_{\xi i}(x)+\epsilon _{\xi i_{2}}\\ \end{aligned} \end{aligned}$$

where $\epsilon _{\xi i_{1}}=\epsilon _{\xi i}-{\tilde{W}}^{T}_{\xi i}S_{\xi i}(x)=0(\epsilon _{\xi i})$ is the approximation error by using $\hat{W}^{T}_{\xi i}S_{\xi i}(x)$ and $\epsilon _{\xi i{2}}=0(\epsilon _{\xi i})$ is the practical approximation error of the system dynamics by using the constant NN vector ${\bar{W}}^{T}_{\xi i}S_{\xi i}(x)$, both of which are very small due to the exponential convergence of ${\tilde{W}}^{T}_{\xi i}$. Based on the convergence of the NN weights, the constant vector of neural weights ${\bar{W}}$ given above can be obtained through the following equation,

9 $$\begin{aligned} {\bar{W}}_{i}=mean_{t\in [t_{a},t_{b}]}\hat{W}_{i}(t) \end{aligned}$$

in which $t_{b}>t_{a}>0$ is the time segment referring to a piece of time segment after the convergence process of the HR model and “mean” is the arithmetic mean.

In the literature of Lyapunov-based system identification, the convergence of system state and the boundedness of the NN weight estimates can be easily achieved, that is, the estimation error of system state ${\tilde{x}}=\hat{x}-x$ will converge to a small neighborhood of zero. However, to achieve accurate identification of system dynamics of the unknown HR model, the boundedness of the NN weight estimates only is far from enough. It is necessary to ensure the convergence of the NN weights, which cannot be achieved unless the PE condition is satisfied. Since the HR system under different parameter possesses recurrent property as discussed above, both the state estimation errors ${\tilde{x}}$ and the NN weight estimation errors ${\tilde{W}}$ do converge exponentially to small neighborhoods of zero when taking the state of the HR model as the input of the RBF neural networks (Wang et al. 2009). Besides, a locally accurate approximation of the unknown dynamics $f_i(x;\mu )$ of the HR system to the desired error is achieved along the recurrent trajectory $\varphi _{\xi }(x_{0})$. The accurate identification of the unknown dynamics and topologies of the HR system can provide more incentives and possibilities for biological experiments and medical treatment as well as other related clinical researches, such as the quantifying and explaining of neurobiological mechanism of neurologic diseases.

## Rapid recognition via Synchronization of the HR model

According to the learning approach, the unknown system topologies as well as the system dynamics of the HR model can be accurately identified and represented in a time-invariant way through constant RBF networks due to the convergence of system parameters. In this section, the obtained system dynamics are further utilized for the rapid recognition of the unknown HR model without identification of its dynamics.

Traditionally, to achieve recognition of a test pattern from a set of training patterns, one possible way is to identify the system dynamics and represent the test pattern by a constant RBF network, and then compare the corresponding NN approximations with that of the training dynamical patterns. But this is a time-consuming process. In this part, the rapid recognition of a test dynamical pattern is considered by using the constant RBF networks achieved in the identification phase. That is, for a given test pattern, a dynamical recognition error system is constructed in a simple disturbed linear time-invariant (LTI) way, which consists the system generating the test pattern and the RBF dynamical model corresponding to one of the training patterns. Thus, the problem of dynamical pattern recognition is turned into a problem of stability and convergence of a recognition error system. The constant RBF networks can quickly recall the learned knowledge by providing accurate approximation to the previously learned system dynamics of a training pattern. To be more specific, a kind of internal and dynamical matching of system dynamics of the test and training pattern proceeds in the recognition error system without re-identifying the system dynamics of the test pattern, during which the numerical computation process of comparing the system dynamics of the corresponding dynamical patterns is naturally eliminated. From this point of view, the process of rapid recognition is completely a dynamical process with knowledge utilization. This is the key to the method of rapid recognition. In addition, the word “rapid” is also reflected in that the recognition process takes place from the beginning of measuring the state of the test pattern without feature extraction from the test pattern, which is normally required in existing neural networks and statistical approaches for static pattern recognition problem.

Rapid recognition of different HR patterns generated from the HR system is of great significance for early diagnosis, classification and control (treatment) of neurologic diseases, such as Parkinson’s and epilepsy. For some patients, rapid and early diagnosis is a matter of life and death. For pattern recognition, a similarity definition based on system dynamics are introduced firstly.

That is, considering the dynamical pattern $\varphi _{\xi }$ generated from the dynamical HR system shown in Eq. (2) and the pattern $\varphi _{\varsigma }$ generated from the HR system under different parameters given as follows,

10 $$\begin{aligned} {\dot{x}}=F^{\prime }(x;\mu ^{\prime }),x(t_{0})=x_{\varsigma 0}, \end{aligned}$$

both of which possesses recurrent characteristic.

The dynamical differences between the corresponding patterns are denoted as $\varDelta f_{i}$. Then, pattern $\varphi _{\varsigma }$ is though to be similar to pattern $\varphi _{\xi }$ if the dynamical difference $\varDelta f_{i}$ is small or it will converge to some neighbourhood of zero ($\varDelta f_{i} \le \epsilon _{\varsigma i}$, in which $\epsilon _{\varsigma i}$ is a positive constant close to zero). From the viewpoint of system state, the smaller the similarity error is, the closer the system state trajectory of pattern $\varphi _{\varsigma }$ is to the system trajectory of pattern $\varphi _{\xi }$ (Wang et al. 2007).

For the dynamical difference $\varDelta f_{i}$, there are many calculation methods. Considering the system characteristics, the following $L_{p}$ function norm is introduced, that is,

11 $$\begin{aligned} \Vert \varDelta f_{i}\Vert _{p}={\int _{t_{0}}^{t_{0}+t}|f_{i}(x;\mu )-f_{i}^{\prime }(x;\mu ^{\prime })|^{p}dt}^{\frac{1}{p}},i=1,\ldots ,n, \end{aligned}$$

in which $t_{0}$ is the initial time and *p* is usually taken as 1 or 2.

According to the similarity definition given above, the similarity measure can only be accurately calculated or quantified if the system dynamics of both patterns are available from measurement, which is usually difficult to achieve for actual systems. But the identification achievements obtained above makes the problem of similarity measure simple and feasible. To be specifically, since the unknown system dynamics $f_{i}(x;\mu )(i=1,\ldots ,n)$ of pattern $\varphi _{\xi }$ can be locally accurately identified and represented through constant RBF networks ${\bar{W}}_{i}^{T}S_{i}(x)(i=1,\ldots ,n)$, the similarity measure between pattern $\varphi _{\xi }$ and pattern $\varphi _{\varsigma }$ can be described in the following way: dynamical pattern $\varphi _{\varsigma }$ is recognized to be similar to pattern $\varphi _{\xi }$, if the distance or error $\varDelta f_{Ni}=|{\bar{W}}_{i}^{T}S_{i}(x)-f^{\prime }_{i}(x;\mu ^{\prime })|$ is small. Considering that:

12 $$\begin{aligned} \begin{aligned} \varDelta f_{N i}&=|{\bar{W}}_{i}^{T}S_{i}(x)-f^{\prime }_{i}(x;\mu ^{\prime })|\\&= |{\bar{W}}_{i}^{T}S_{i}(x)-f_{i}(x;\mu )+f_{i}(x;\mu )-f^{\prime }_{i}(x;\mu ^{\prime })|\\&\le |{\bar{W}}_{i}^{T}S_{i}(x)-f_{i}(x;\mu )|+|f_{i}(x;\mu )-f^{\prime }_{i}(x;\mu ^{\prime })|\\&\le \epsilon _{\xi i_{2}}+\epsilon _{\varsigma i} \end{aligned} \end{aligned}$$

in which $\epsilon _{\xi i_{2}}$ is the approximation error given in Eq. (8) and $\epsilon _{\varsigma i}$ is a finite positive constant. If the error $\epsilon _{\xi i_2}+\epsilon _{\varsigma i}$ comes small under the condition that the unknown dynamics of system $f(x;\mu )$ being accurately identified, the positive constant $\epsilon _{\varsigma i}$ can be very small. That is, the corresponding pattern $\varphi _{\varsigma }$ is similar to pattern $\varphi _{\xi }$. Meanwhile, the dynamical differences $\varDelta f_{N i}$ can be calculated through the $L_{p}$-norm, that is,

13 $$\begin{aligned} \Vert \varDelta f_{N i}\Vert _{p}=\left( \frac{1}{t}\int _{t_{0}}^{t_{0}+t}|{\bar{W}}_{i}^{T}S_{i}(x)-f_{i}^{\prime }(x;\mu ^{\prime })|^{p}dt\right) ^{\frac{1}{p}},i=1,\ldots ,n \end{aligned}$$

in which $t_{0}$ is the initial time and *p* is set as 1 or 2 usually.

It can be seen from the similarity definition that both of the system state and the nonlinear dynamics of the two patterns are related to the similarity measure. The local accurate identification of the unknown system dynamics is the premise for accurate similarity calculation. Furthermore, the similarity definition mentioned above gives a feasible and quantifiable method to calculate the similarity error between different systems.

Based on the similarity definition, we further discuss the recognition process of the HR model. Consider a training set containing dynamical HR pattern $\varphi ^{k}_{\xi } (k=1,\ldots ,M)$ generated from the system

14 $$\begin{aligned} {\dot{x}}=F^{k}(x;\mu ^{k}), x(t_{o})=x^{k}_{\xi 0}, \end{aligned}$$

in which $\mu ^{k}$ is the parameter vector and the system possesses recurrent characteristics under different parameter value. According to the identification process, the unknown system dynamics $F^{k}(x;\mu ^{k})=[f_{1}^{k}(x;\mu ^{k}),\ldots ,f_{n}^{k}(x;\mu ^{k})]^{T}$ of the HR system has been locally accurately identified and stored in the constant RBF networks given as ${\bar{W}}^{k^{T}}S(x)=[{\bar{W}}_{1}^{k^{T}}S_{1}(x),\ldots ,{\bar{W}}_{n}^{k^{T}}S_{n}(x)]^{T}$.

Then, for the $kth (k=1,\ldots ,M)$ training pattern $\varphi ^{k}_{\xi }$ generated from the HR system (14), a dynamical model is constructed by using the time-invariant representation ${\bar{W}}^{kT}S(x)$:

15 $$\begin{aligned} \dot{{\bar{x}}}^{k}=-{\bar{B}}({\bar{x}}^{k}-x)+{\bar{W}}^{kT}S(x), \end{aligned}$$

where ${\bar{x}}^{k}=[{\bar{x}}_{1}^{k},\ldots ,{\bar{x}}_{n}^{kT}]$ is the state of the dynamical model, *x* is the state of an input test pattern $\varphi _{\varsigma }$ generated from Eq. (10) with $x(t_{0})=x_{\varsigma 0}$ been the initial state. ${\bar{B}}=diag\{{\bar{b}}_{1},\ldots ,{\bar{b}}_{n}\}$ is a diagonal matrix which is adjustable small value and kept as the same for all training patterns. Furthermore, for the given test pattern $\varphi _{\varsigma }$ and the training pattern $\varphi ^{k}_{\xi }$, the following recognition error system is obtained:

16 $$\begin{aligned} \dot{{\tilde{x}}}_{i}^{k}=-{\bar{b}}_{i}{\tilde{x}}_{i}^{k}+\left( {\bar{W}}_{i}^{kT}S_{i}(x)-f^{^{\prime }}_{i}(x;\mu ^{\prime })\right) ,i=1,\ldots ,n \end{aligned}$$

where ${\tilde{x}}_{i}^{k}={\bar{x}}_{i}^{k}-x_{i}$ is the synchronization error. Then, considering the Lyapunov function $V_{i}=\frac{1}{2}{\tilde{x}}_{i}^{2}$, its derivation is achieved according to the error system shown in Eq. (16), that is:

17 $$\begin{aligned} {\dot{V}}_{i}={\tilde{x}}_{i}\dot{{\tilde{x}}}_{i}=-{\bar{b}}_{i}{\tilde{x}}^{2}_{i}+{\tilde{x}}_{i}\left( {\bar{W}}_{i}^{T}S_{i}(x)-f_{i}^{\prime }(x;\mu ^{\prime })\right) . \end{aligned}$$

By using the scaling principle, we have

18 $$\begin{aligned} \begin{aligned} {\dot{V}}_{i}&=-\frac{{\bar{b}}_{i}}{2}{\tilde{x}}_{i}^{2}-\frac{{\bar{b}}_{i}}{2}{\tilde{x}}_{i}^{2}+{\tilde{x}}_{i} \left( {\bar{W}}^{T}_{i}S_{i}(x)-f^{\prime }_{i}(x;\mu ^{\prime })\right) \\&=-\frac{{\bar{b}}_{i}}{2}{\tilde{x}}_{i}^{2}-\frac{{\bar{b}}_{i}}{2}\left[ {\tilde{x}}_{i}-\frac{\left( {\bar{W}}_{i}^{T}S_{i}(x)-f^{\prime }_{i}(x;\mu ^{\prime })\right) }{{\bar{b}}_{i}}\right] ^{2}\\&\quad +\frac{\left( {\bar{W}}_{i}^{T}S_{i}(x)-f^{\prime }_{i}(x;\mu ^{\prime })\right) ^{2}}{2{\bar{b}}_{i}},\\&\le -\frac{{\bar{b}}_{i}}{2}{\tilde{x}}_{i}^{2}+\frac{|{\bar{W}}_{i}^{T}S_{i}(x)-f^{\prime }_{i}(x;\mu ^{\prime })|^{2}}{2{\bar{b}}_{i}}\\ \end{aligned} \end{aligned}$$

in which,$|{\bar{W}}_{i}^{T}S_{i}(x)-f^{\prime }_{i}(x;\mu ^{\prime })|\le \epsilon _{i2}$ is the identification error as can be seen from Eq. (8). For the sake of illustration, denote $\rho _{i}=\frac{|{\bar{W}}_{i}^{T}S_{i}(x)-f^{\prime }_{i}(x;\mu ^{\prime })|^{2}}{2{\bar{b}}_{i}}$.

Then, bring $V_{i}$ into the above equation, there is,

19 $$\begin{aligned} \begin{aligned} {\dot{V}}_{i}&\le -\frac{{\bar{b}}_{i}}{2}{\tilde{x}}_{i}^{2}+\frac{|{\bar{W}}_{i}^{T}S_{i}(x)-f^{\prime }_{i}(x;\mu ^{\prime })|^{2}}{2{\bar{b}}_{i}}\\&= -{\bar{b}}_{i}V_{i}+\rho _{i}, \end{aligned} \end{aligned}$$

Then, the solution of the differential inequality can be obtained, that is,

20 $$\begin{aligned} 0 \le V_{i}(t)< \rho _{i}+(V_{i}(0)-\rho _{i})exp(-{\bar{b}}_{i}t). \end{aligned}$$

which means that the synchronization error satisfies the following inequality,

21 $$\begin{aligned} {\tilde{x}}_{i}^{2}<2\rho _{i}+2V_{i}(0)exp(-{\bar{b}}_{i}t). \end{aligned}$$

Based on the accurate identification of the training pattern $\varphi ^{k}_{\xi }$ obtained in “Section Identification of the HR model via deterministic learning theory”, the synchronization errors ${\tilde{x}}_{i}^{k}(i=1,\ldots ,n)$ between the test pattern and training patterns will converge exponentially to a neighbourhood of zero. More specifically, the synchronization error $|{\tilde{x}}_{i}|_{t\ge T}$ is approximately proportional to the difference between the system dynamics $f^{\prime }_{i}(x;\mu ^{\prime })$ of the test pattern $\varphi _{\varsigma }$ and the identified system dynamics ${\bar{W}}_{i}^{kT}S_{i}(x)$ of the training pattern $\varphi _{\xi }^{k}$. Furthermore, the synchronization error ${\tilde{x}}_{i}^{k}(t)$, which depicts and measures the dynamic difference between the test pattern and the training pattern, can also be calculated through the average 1-norm, that is:

22 $$\begin{aligned} \Vert {\tilde{x}}_{i}^{k}(t)\Vert _{1}=\frac{1}{t}|{\tilde{x}}_{i}^{k}(t)|dt, i=1,\ldots ,n. \end{aligned}$$

For dynamical patterns generated from the HR system under different parameter values, it is difficult to measure the differences or similarities of the patterns since the dynamic properties of the systems themselves are unknown to us when considering the complexity of the HR system itself and the changeable dynamic environment. The local accurate identification of the unknown system dynamics of the HR model obtained in the identification part plays a key role for pattern recognition. And more importantly, the time-invariant and spatially distributed representation of the identified system dynamics further ensures the rapid recognition of the unknown test pattern generated from the HR system. To be specific, the stored training model is directly used for pattern recognition, which saves the time of re-learning of the unknown dynamics of the test pattern. According to the recognition error system (16), the synchronization error $|{\tilde{x}}_{i}|$ is naturally considered as the recognition error, namely the similarity error between the test pattern and the training patterns. The significance of this work lies in that rapid recognition of different HR patterns will lay foundation for early rapid diagnosis of neurologic diseases, such as Parkinson’s and epilepsy, and buy effective time for timely treatment and control of disease progression.

## Numerical simulations

### Identification of the HR model under different parameters

In this part, the identification of unknown dynamics of the HR model via deterministic learning algorithm is demonstrated. All the parameters except for *I* are fixed as constants, that is $a=1.0, b=3.0, c=1.0, d=5.0, r=0.005, s=4.0, q=-1.6$. The membrane input current *I* is taken as the control parameter varying from 1.5 to 4.

For the convenience of presentation, just take the subsystem $f_2(x;\mu )=c-dx^2-y$ of the HR model given in Eq. (1) as an example to show the identification effect. Construct the centers of the RBF networks $\hat{W}_2^TS_2(x)$ evenly placed on $[-2,2]\times [-15,6]$, and the widths of the RBF NN are given as $\eta _2=1$. The weights of the RBF networks are updated online according to Eq. (5), in which the design parameters are $\varGamma _2=5, \sigma _2=0.0001, {\bar{a}}_{i}=2$. The initial weight is $\hat{W}_2(0)=0.0$, and the initial condition of the state vector of system (3) are set as $[x_1(0),x_2(0),x_3(0)]^T=[1,-1,2]^T$ and $[\hat{x}_1(0),\hat{x}_2(0),\hat{x}_3(0)]^T=[2,-0.3,2]^T$.

**Fig. 5:**
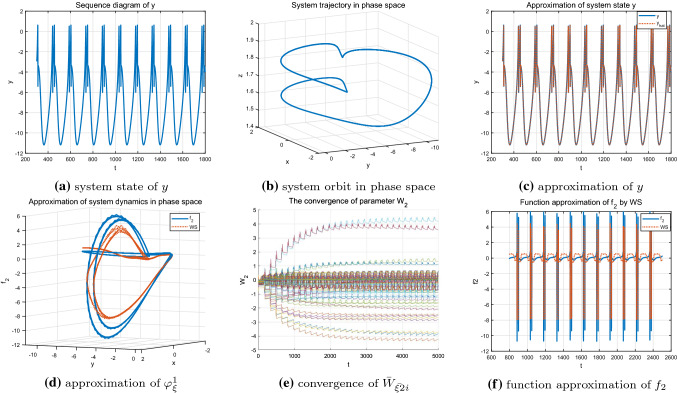
Identification of the HR model $\varphi _{\xi }^1$ with $I=1.8$

Firstly, the regular bursting (period-doubling discharge) state under parameter $I=1.8$ of the HR model is identified. The time series of the system state *y* shown in Fig. 5a, b are well approximated by using the localized RBF networks, which can be seen from Fig. 5c. Figure 5e demonstrates the partial parameter convergence during the learning process. That is, only the neurons whose centers located close to the system orbit can be activated and updated, and the corresponding neuron weight estimates will converge to their optimal values $W^{*}_{\xi i}$. Those neurons that located far away from the system trajectory can not be activated and updated, thus, their weight estimates whose initial values are zero will keep zero unchanged. Under the condition of the convergence of neuron weights, the ideal NN approximations of the unknown system dynamics $f_{2}(x;\mu )$ along the system trajectory are achieved and shown in Fig. 5d, f.

**Fig. 6:**
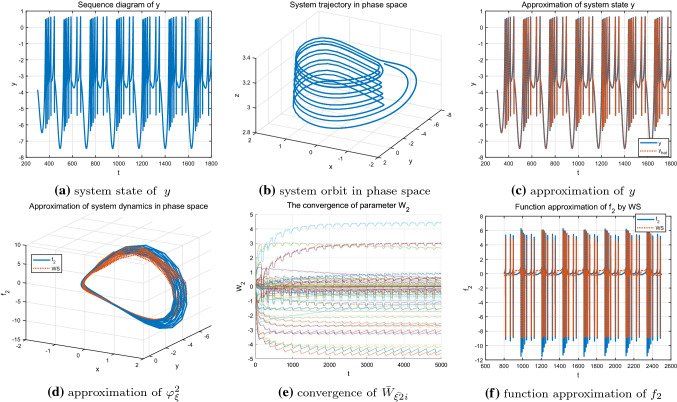
Identification of the HR model $\varphi _{\xi }^2$ with $I=3.2$

Secondly, by setting $I=3.2$, the HR system presents chaotic state and the corresponding system state and state trajectory are shown in Fig. 6a, b, respectively. From Fig. 6c, it is shown that even the chaotic state carries more abundant dynamic information, the system state also can be well approximated by using the local redial basic function neural networks. Furthermore, more neurons are involved and activated, and the corresponding neuron weights do converge to their ideal values as demonstrated in Fig. 6e. Thus, the unknown system dynamics $f_{2}(x;\mu )$ is locally accurately approximated along the chaotic orbit, which can be seen from the Fig. 6d, f.

**Fig. 7:**
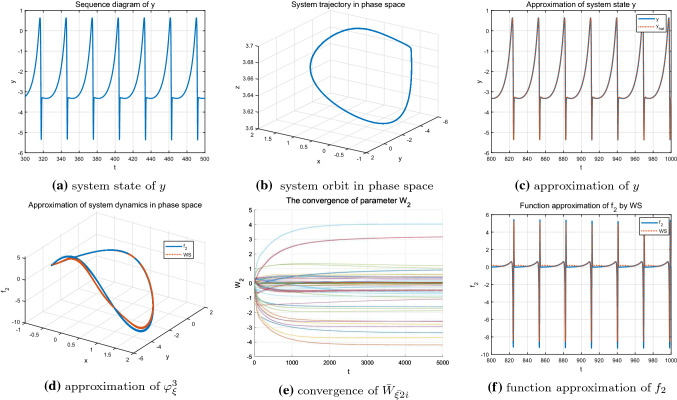
Identification of the HR model $\varphi _{\xi }^3$ with $I=3.6$

Finally, the single-cycle bursting state of the system under parameter $I=3.6$ shown in Fig. 7a, b is considered. At this time, the system exhibits a more regular periodicity and contains less information compared with chaotic state. Since the input trajectory of the system is relatively regular and simple, only some neurons are activated and the weights of the other neurons remain zero. Figure 7e shows the convergence process of the neural weight estimates and further reveals the above description. The accurate identification of the state *y* and the unknown system dynamics $f_{2}(x;\mu )$ are achieved (see Fig. 7c, d, f).

Besides parameter *I*, *r* is also an important control parameter of HR system, which is related to the calcium concentration. Different value of *r* can make different bursting patterns and the change of calcium concentration may affects the normal function of neurons to some extent. Thus, the identification of the system topology under control parameter *r* is of great significance.

In this part, the parameter *r* is taken as the control parameter with parameter *I* being set to 3. Then, the identification of the topology of the HR system under parameter *r* is discussed. All the parameters are fixed as the same constant given above, that is $a=1.0, b=3.0, c=1.0, d=5.0, s=4.0, q=-1.6$. The value of control parameter *r* is ranging from 0 to 0.05. The subsystem $f_2(x;\mu )=c-dx^2-y$ of the HR model is also taken as an example to show the identification effect for the convenience of description. The centers of the RBF networks $\hat{W}_2^TS_2(x)$ are evenly placed on $[-2,2]\times [-15,6]$, and the widths of the RBF NN $\eta _2=1$. The weights of the RBF networks are updated online according to Eq. (4), in which the design parameters are $\varGamma _2=3,\sigma _2=0.0001$. The initial weight is $\hat{W}_2(0)=0.0$, and the initial condition of the system given in Eq. (3) are set as $[x_1(0),x_2(0),x_3(0)]^T=[0.1,-0.1,3]^T$ and $[\hat{x}_1(0),\hat{x}_2(0),\hat{x}_3(0)]^T=[0.2,0.3,2]^T$.

**Fig. 8:**
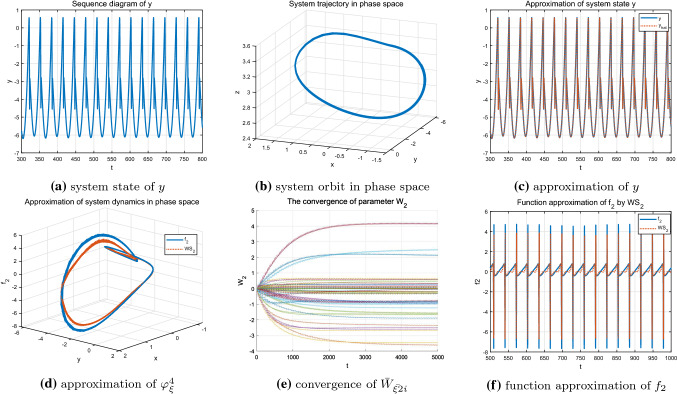
Identification of the HR model $\varphi _{\xi }^4$ with $r=0.045$

**Fig. 9:**
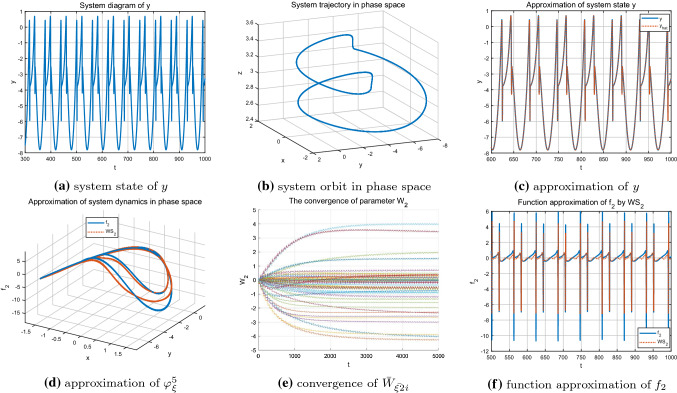
Identification of the HR model$\varphi _{\xi }^5$ with $r=0.025$

**Fig. 10:**
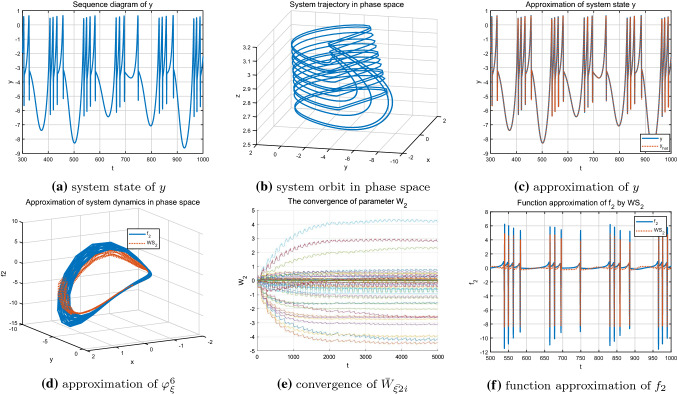
Identification of the HR model $\varphi _{\xi }^6$ with $r=0.006$

As shown in Figs. 8, 9 and 10, under different parameter *r*, accurate identification of three different nonlinear dynamic states including regular bursting (simple periodic) state and complex chaotic bursting state of the HR model are obtained through DL algorithm. In addition, the locally convergence of neuron weight estimates are clearly demonstrated in Figs. 8e, 9e and 10e, that is, the neurons located close to the system trajectory are activated and the corresponding weights converge to their ideal values while the weight estimates are almost zero for those neurons that far away from the system trajectory. In addition, whether the system is in a simple and regular periodic state (as shown in Figs. 8a, b, 9a, b) or a complex chaotic state (as shown in Fig. 10a, b), the system states as well as the unknown system dynamics can be well approximated along the system trajectory.

**Fig. 11:**
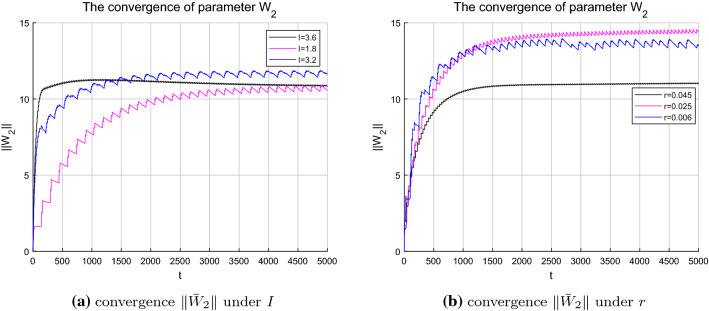
Parameter convergence $\Vert {\bar{W}}_{2}\Vert$ under different parameter condition

Further considering the weight convergence of the identification process for the HR system under different parameter *I* and *r* as shown in Fig. 11a, b, it is noticed that when $I=3.6$ and $r=0.045$ which correspond to regular single-cycle bursting state, the neuron weight estimates converge faster compared to that of the complex chaotic state under the condition that $I=3.2$ and $r=0.006$. This is because the more complex the system trajectories are, the more neurons are needed to be involved and activated for the identification process, and the minimum amount of time for the orbit to pass through the neuron centers in certain areas might get longer. In addition to the rate of convergence, the convergence process is relatively smooth for the system in simple periodic state, that is, the weight estimates $\hat{W_{\xi i}}$ converges to the ideal weights $W^{*}_{\xi i}$ without bias; whereas, when the system is in a complex chaotic state, the corresponding convergence process experienced some fluctuation, but it could also converge to the neighborhood of the ideal weights within a certain time. The convergence of the neuron weight estimates ensures the accurate identification of the unknown system dynamics and topologies.

The identification results clearly demonstrate that although the complex HR system contains diverse dynamic behaviors under different system parameters, different system states as well as the nonlinear topologies can still be well identified along the system trajectory in a deterministic way. Furthermore, the more simple the dynamic properties of the HR system, the more stable and faster the process of convergence, as shown in Fig. 11a, b.

### Rapid pattern recognition of the HR model

In this section, the rapid recognition algorithm is verified by simulations. The dynamical patterns $\varphi _{\xi }^{4,5,6}$ (with parameter $r=0.045, 0.025, 0.006$, respectively) mentioned in the identification “section Identification of the HR model under different parameters” are considered as the training patterns, which corresponds to simple periodic patterns and chaotic pattern, respectively. The time-invariant representations ${\bar{W}}^{kT}S(x)(k=4,5,6)$ of the training patterns $\varphi _{\xi }^{4,5,6}$ obtained in the identification process are introduced for pattern recognition. That is, for the given test patterns $\varphi _{\varsigma }^{k^{\prime }} (k^{\prime }=1,2,3)$, the dynamical recognition models are constructed as follows,

23 $$\begin{aligned} \dot{{\tilde{x}}}_{i}^{kk^{\prime }}=-{\bar{b}}_{i}{\tilde{x}}_{i}^{kk^{\prime }}+\left( {\bar{W}}_{i}^{kT}S_{i}(x)-f^{\prime k^{\prime }}_{i}(x;\mu ^{\prime })\right) ,i=1,\ldots ,n, \end{aligned}$$

where ${\tilde{x}}^{kk^{\prime }}_{i}$ is the synchronization error of state *x* for training pattern $\varphi _{\xi }^{k}(k=4,5,6)$ and test pattern $\varphi _{\varsigma }^{k\prime }(k^{\prime }=1,2,3)$, $b_i$ is a design constant which is set as 5 in this part.

To verify the rapid recognition approach, test pattern $\varphi _{\varsigma }^{1}$, $\varphi _{\varsigma }^{2}$ and $\varphi _{\varsigma }^{3}$ are generated from Eq. (10) under different value of *r*. In detail, the initial condition and the system parameters are chosen as $x(0)=[x_{1}(0),x_{2}(0),x_{3}(0)]^{T}=[0.1,-0.2,0.3]^{T}$. Besides, the parameter $r^{\prime }$ is chosen as 0.043, 0.027, 0.005 for test patterns $\varphi _{\varsigma }^{1}$, $\varphi _{\varsigma }^{2}$ and $\varphi _{\varsigma }^{3}$, respectively.

**Fig. 12:**
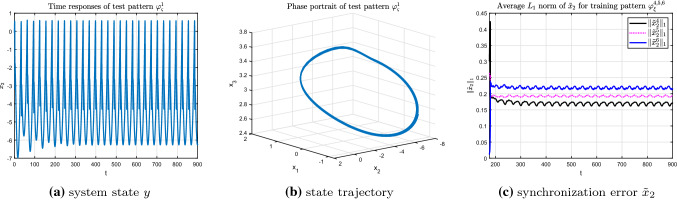
Recognition of test pattern $\varphi _{\varsigma }^{1}$ (r = 0.043)for training patterns $\varphi _{\xi }^{4,5,6}$

Firstly, consider the recognition process of test pattern $\varphi _{\varsigma }^{1}$ by using the given training patterns $\varphi _{\xi }^{4,5,6}$, in which, pattern $\varphi _{\xi }^{4,5}$ correspond to regular periodic bursting modes and $\varphi _{\xi }^{6}$ is the representative of complex chaotic model. As can be intuitively seen from Fig. 12a, b, both the time response of state *y* and the state trajectory $\varphi _{\varsigma }^{1}(x_{0})$ of the test pattern $\varphi _{\varsigma }^{1}$ possess single period characteristic. The average $L_{1}$ norms of the state synchronization error $\Vert {\tilde{x}}_{2}^4\Vert _{1}$ between the test pattern $\varphi _{\varsigma }^{1}$ and training pattern $\varphi _{\xi }^{4}$ is much smaller than that of the training patterns $\varphi _{\xi }^{5,6}$. Besides, the discrimination of the synchronization errors is obvious as shown in Fig. 12c. That is, test pattern $\varphi _{\varsigma }^{1}$ is recognized to be more similar to training pattern $\varphi _{\xi }^{4}$ than to training patterns $\varphi _{\xi }^{5,6}$.

**Fig. 13:**
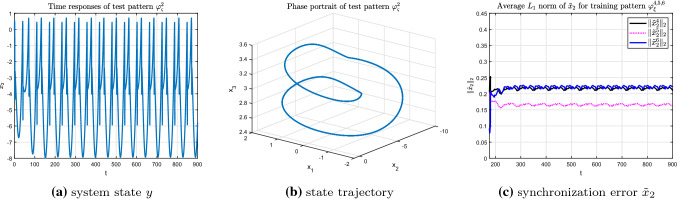
Recognition of test pattern $\varphi _{\varsigma }^{2}$ (r = 0.027) for training patterns $\varphi _{\xi }^{4,5,6}$

Similarly, for test pattern $\varphi _{\varsigma }^{2}$, which is a regular doubling-periodic model which contains more information compared with the test pattern $\varphi _{\varsigma }^{1}$ (see Fig. 13a, b), the corresponding synchronization error shown in Fig. 13c demonstrates that test pattern $\varphi _{\varsigma }^{2}$ is more similar to the training pattern $\varphi _{\xi }^{5}$. Meanwhile, the synchronization error between test pattern $\varphi _{\varsigma }^{2}$ and training patterns $\varphi _{\xi }^{4,6}$ are so close that it is difficult to distinct which one is smaller. This result is in good agreement with the nonlinear characteristics of the HR model, and further verifies the effectiveness of the recognition algorithm.

**Fig. 14:**
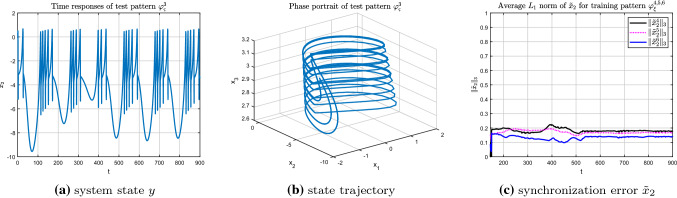
Recognition of test pattern $\varphi _{\varsigma }^{3}$ (r = 0.005) for training patterns $\varphi _{\xi }^{4,5,6}$

Finally, the chaotic bursting model under $r=0.005$ is taken as the test pattern $\varphi _{\varsigma }^{3}$. From the synchronization error shown in Fig. 14c, it can be seen clearly that among the synchronization errors, the error $\Vert {\tilde{x}}_{2}^6\Vert _{3}$ of the chaotic pattern $\varphi _{\xi }^{6}$ is the smallest compared to that of the regular bursting model $\varphi _{\xi }^{4}$ and $\varphi _{\xi }^{5}$. It means that test pattern $\varphi _{\varsigma }^{3}$ is more similar with the training pattern $\varphi _{\xi }^{6}$, both of which are complex chaotic models.

## Conclusion and discussion

In this paper, we mainly focused on the problem of topology identification and the rapid recognition of the HR neural networks under unknown dynamical environment. Firstly, the unknown system dynamics and the nonlinear topologies of the HR model under different control parameters have been numerically analyzed. The results indicate that the HR system possesses abundant dynamic behaviors and complex changeable topological structures. Then, the identification of the dynamic behaviors and system topologies of the HR model under unknown dynamic environment via DL algorithm have been discussed. The parameter *I* which represents the membrane input current and the parameter *r* that influences the calcium concentration have been taken as the control variables respectively. By using the DL algorithm, all the dynamic behaviors have been locally accurately identified through appropriately laying out the neural networks and adjusting the learning parameters. The successful identification of the HR system is helpful to understand the dynamic process and mechanism of the neurologic system in a more comprehensive and profound way. This achievement can be used for further researches such as quantifying and explaining biological mechanism as well as predicting physiological phenomena of neurologic diseases. Additionally, based on the identification achievement, rapid recognition of different dynamical patterns generated from HR system through synchronization have been achieved. This achievement can help lay the foundation for early and rapid diagnosis of neurologic diseases, such as Parkinson’s and epilepsy, and will win over time for timely treatment and control of disease progression. Simulations have demonstrated the identification and recognition effects.

## Data Availability

Data sharing not applicable to this article as no datasets were generated or analysed during the current study.
